# Quantitative Proteomic Analysis Provides Insights into Rice Defense Mechanisms against *Magnaporthe oryzae*

**DOI:** 10.3390/ijms19071950

**Published:** 2018-07-03

**Authors:** Siyuan Lin, Pingping Nie, Shaochen Ding, Liyu Zheng, Chen Chen, Ruiying Feng, Zhaoyun Wang, Lin Wang, Jianan Wang, Ziwei Fang, Shaoxia Zhou, Hongyu Ma, Hongwei Zhao

**Affiliations:** 1 College of Plant Protection, Nanjing Agricultural University, Nanjing 210095, China; 2016102048@njau.edu.cn (S.L.); sdniepingping@163.com (P.N.); 2017102050@njau.edu.cn (S.D.); 2017102052@njau.edu.cn (L.Z.); 2016102047@njau.edu.cn (C.C.) 2016802185@njau.edu.cn (R.F.); 2014202018@njau.edu.cn (Z.W.); 2015202020@njau.edu.cn (L.W.); 12115104@njau.edu.cn (J.W.); 12115106@njau.edu.cn (Z.F.); sxzhou@njau.edu.cn (S.Z.); 2 Key Laboratory of Integrated Management of Crop Diseases and Pests, Nanjing Agricultural University, Ministry of Education, Nanjing 210095, China; 3 College of Life Sciences, Zaozhuang University, Zaozhuang 277160, China

**Keywords:** innate immunity, basal defense, rice blast, *Magnaporthe oryzae*, proteomics, iTRAQ

## Abstract

Blast disease is one of the major rice diseases, and causes nearly 30% annual yield loss worldwide. Resistance genes that have been cloned, however, are effective only against specific strains. In cultivation practice, broad-spectrum resistance to various strains is highly valuable, and requires researchers to investigate the basal defense responses that are effective for diverse types of pathogens. In this study, we took a quantitative proteomic approach and identified 634 rice proteins responsive to infections by both *Magnaporthe oryzae* strains Guy11 and JS153. These two strains have distinct pathogenesis mechanisms. Therefore, the common responding proteins represent conserved basal defense to a broad spectrum of blast pathogens. Gene ontology analysis indicates that the “responding to stimulus” biological process is explicitly enriched, among which the proteins responding to oxidative stress and biotic stress are the most prominent. These analyses led to the discoveries of OsPRX59 and OsPRX62 that are robust callose inducers, and OsHSP81 that is capable of inducing both ROS production and callose deposition. The identified rice proteins and biological processes may represent a conserved rice innate immune machinery that is of great value for breeding broad-spectrum resistant rice in the future.

## 1. Introduction

Rice is the staple food that feeds about one third of the world population. Rice blast disease is one of the major diseases threatening rice production, which is estimated to cause about 30% annual yield loss [1]. Due to its enormous economic importance, studying the interaction between rice and the causal agent of the rice blast disease, *Magnaporthe oryzae*, is of great scientific and economic significance. Also, as more and more research has been focused on the mechanism governing this mutual interaction, the rice-*M. oryzae* system has become a model system of cereal plants and their fungal pathogens [2]. The recently discovered *M. oryzae* colonization of wheat [3] strengthened its significance.

Plants are capable of defending themselves against various pathogens. The plant innate immune system is composed of multiple components located at both the plasma membrane and inside the cells. The receptors on the membrane sense the pathogen-associated molecular patterns (PAMP) and initiate the PAMP-triggered immunity (PTI). Due to the extreme conserved nature of PAMPs, PTI is constitutionally effective against broad-spectrum pathogens, which is characterized by the rapid launch of reactive oxygen species (ROS) and callose deposition around the infection loci [4]. Some pathogens win the combat over PTI, most of which through secreting effectors that specifically interrupt PTI. Plants have evolved machinery that recognizes effectors and activates effector-triggered immunity (ETI). ETI is usually associated with massive gene expression reprogramming, including activation of defense-related genes, alteration of cellular redox status, and activation of phytohormone signaling pathways such as salicylic acid (SA) and jasmonic acid (JA) [5].

To breed blast-resistant rice lines that are effective for various strains in the field, we need understand the common mechanism that rice employs to sense the diverse type of blast pathogens, to identify the signals that are passed downstream, and find out defense responses that are activated and are efficient to restrain the progression of the disease. Previous effort discovered critical immune modules that are important for rice defense responses against the blast disease, such as the enhanced ability of ROS production and callose deposition [4], activation of the mitogen-activated protein kinase (MAPK) signaling cascade [6], and preferential employment of the SA or JA signaling pathways [7]. However, most of our knowledge is from genetic examinations, which lack interpretation from a proteomic view. Moreover, essential genes playing primary defensive roles to a specific strain are over-emphasized, with molecules and biological processes responding to broad-spectrum resistance overlooked.

Proteome is the entire set of proteins expressed by a genome, cell, tissue, or organism at a certain time. By comparing the proteomic profiles between mock- and *M. oryzae*-treated rice seedlings or suspension cultured cells, critical rice immune components against blast disease were identified [8,9]. This was particularly facilitated by the tandem utilization of high-performance liquid chromatography (HPLC) and mass spectrometry (MS) over the past two decades. By virtue of the superb fractionation capability of HPLC and excellent sensitivity of MS, high throughput proteomic profiling has emerged as a powerful tool to investigate the protein machineries involved in blast disease resistance at trace amount-level [10,11].

In this study, we employed an isobaric tag for relative and absolute quantitation (iTRAQ) technique that can compare protein expression levels between different rice samples. We focused on rice proteins responding to both the virulent (Guy11) and the avirulent (JS153) *M*. *oryzae* strains, but not just to any one of them. We aimed to identify the conserved basal defensive components of broad-spectrum blast disease pathogens. By applying the iTRAQ method, we found that both Guy11 and JS153 typically induce proteins involved in biological processes such as “responses to oxidative stress” (such as OsAPX1, OsPRX59, and OsPRX62) and “response to biotic stress” (such as OsHSP81, OsPBZ1, and OsPR10). We further proved that OsPRX59 and OsPRX62 are robust cell wall synthetic enhancers while OsHSP81, OsPBZ1, and OsPR10 participate in both ROS accumulation and callose deposition. Specific expression variation of several SA signaling components was also observed, suggesting its involvement in responding to either Guy11 or JS153 infection. Our discovery identified the critical innate immune machinery that will facilitate breeding of rice with broad-spectrum blast resistance.

## 2. Results

To identify rice proteins that are potentially involved in defense against rice blast disease, we employed the iTRAQ peptide labeling approach and liquid chromatography–tandem mass spectrometry (LC–MS/MS) that can determine the amount of proteins from different sources in a single experiment [12,13]. Total rice (Nipponbare; three-leaf-stage) proteins from both *M. oryzae*-infected (24 and 72 h post inoculation; hpi) and healthy rice (0 hpi) were examined. Both a relative virulent (Guy11) and an avirulent strain (JS153) were used to explore the interaction between rice and the blast pathogens. Guy11 is a relative virulent strain that causes moderate disease symptoms on Nipponbare, which is weaker than on Kongyu 131 but stronger than on Lijiangxintuanheigu (LTH) [14,15]. JS153 is an avirulent strain that shows no disease symptoms on Nipponbare. Therefore, the similarities between Guy11 and JS153 responses represent rice basal defense against blast disease, while the differences represent defense responses initiated by the effector-triggered immunity (ETI) [4]. We examined two independent biological replicates, from which 3109 and 2990 rice proteins were identified, respectively (Table S1). Between these two repeats, 1618 proteins were found in both assays (Table S2), representing reproducible proteins from our quantitative proteomic measurements. Differentially expressed (DE) rice proteins were identified by comparing the protein expression profiles between the *M. oryzae*-treated and the healthy rice (24/0 and 72/0 hpi, respectively). We only selected for further study the proteins that were highly confident (*p* < 0.5), had more than 10% peptide coverage from mass spectrometry, and were either more than 1.25-fold or less than 0.8-fold in the *M. oryzae*-treated samples than in the healthy samples [16]. According to these criteria, we obtained 634 DE-proteins upon *M. oryzae* infection (Table S3).

**Figure 1:**
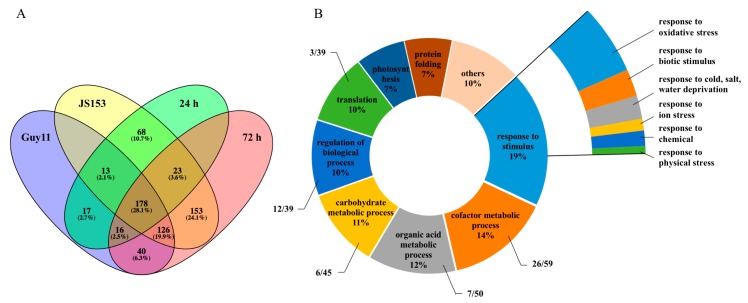
Gene ontology (GO) analysis on differentially expressed (DE)-proteins and their distribution map. (**A**) Venn diagram shows the expression preference of the 634 DE-proteins; (**B**) Gene ontology analysis of the 634 DE-proteins. The pie chart indicates the biological process of 634 DE-proteins. The fan-shaped chart shows the subterms of the 40 DE-proteins responding to stimulus.

We analyzed the 634 DE-proteins according to their expression preferences in different treatments. We found that 390 proteins were explicitly differentially expressed after Guy11 infection, among which 30 proteins expressed only at 24 hpi, and 170 proteins just expressed at 72 hpi, while 194 proteins expressed at both 24 and 72 hpi. After JS153 infection, 561 were specifically differentially expressed (Figure 1A), among which 81 proteins only expressed at 24 hpi, 279 only expressed at 72 hpi, while 201 proteins expressed at both 24 and 72 hpi. These proteins represent distinct rice responses against both the virulent and the avirulent blast strains. Importantly, 317 DE-proteins from both Guy11 and JS153-infected rice, but not from just one of the treatments, were identified, accounting for 50.1% of the total DE-proteins.

To find what biological processes are employed for defense to blast disease by rice, we performed a gene ontology (GO) analysis of these 634 DE-proteins. Six biological processes with more than 10% enrichment were identified. These enriched biological processes were “response to stimulus” (19%), “cofactor metabolic process” (14%), “organic acid metabolic” (12%), “carbohydrate metabolic” (11%), “regulation of biological process” (10%), and “translation” (10%). “Response to stimulus” is composed of proteins related to major biological responses to stimuli such as biotic, abiotic, chemical, and wounding stresses. The specific enrichment of proteins responding to stimulus indicates that after the *M. oryzae* infection, rice makes a considerable adjustment by shifting many biological processes toward palliating and preventing cellular damage by pathogen infection (Figure 1B). “Cofactor metabolic process” refers to chemical reactions and pathways involving a cofactor that is required for the activity of an enzyme or other protein. Our results indicate that 26 out of the 59 cofactors are associated with response to stimulus (Table S4). “Organic acid metabolic” includes chemical reactions and pathways involving acidic compounds containing carbon in covalent linkage. Seven out of the 50 proteins in this category are associated with response to stimulus. We further found that six out of the 45 “carbohydrate metabolic” proteins, 12 out of the 39 proteins belong to the “regulation of biological process”, and three out of the 39 proteins belonging to “translation” are related to “response to stimulus”.

**Figure 2:**
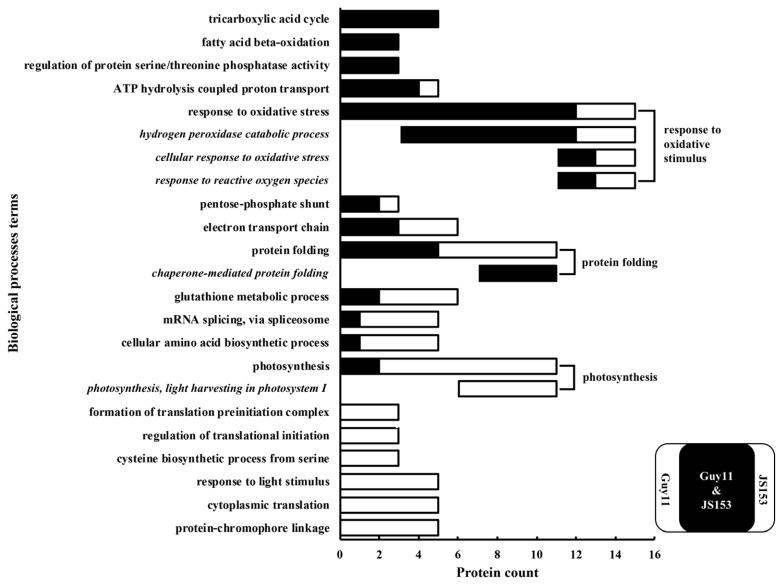
Biological processes (BP) analysis on DE-proteins and their expression preference. The 634 DE-proteins were analyzed by using the DAVID Bioinformatics Resources 6.8. Biological processes were sorted according to their enrichment in both Guy11 and JS153 infections. The terms in italic indicate sub-terms that belong to the major terms above.

When the DE-proteins responding both to Guy11 and JS153 infections were compared to those just responding to either Guy11 or JS153, we found defense-related biological processes were highly enriched (Figure 2). For example, when biological processes (BP) were aligned according to their preferentially differential expression by both Guy11 and JS153 infection, many defense-related biological processes were more enriched, including “regulation of protein serine/threonine phosphatase activity”, “response to oxidative stress”, “protein folding”, and “glutathione metabolic process”. In contrast, other biological processes, such as “mRNA splicing”, “cellular amino acid biosynthesis”, “photosynthesis”, “response to light stimulus”, and “cytoplasm translation” are preferentially enriched in responses just to Guy11 or JS153 infection (Figure 2). The preferential enrichment of defense-related biological processes both in Guy11- and JS153-infected rice indicates that a consensus set of defense responses are allocated to defend both virulent and avirulent blast disease pathogens, whereby we may identify the innate immune components essential for a broad spectrum of blast pathogens.

Based on the fact that defense responses, both to virulent and avirulent infection represent a conserved defense machinery against broad-spectrum blast pathogens, and considering the preferential enrichment of rice proteins related to the “response to stimulus” (Figure 1B), we further narrowed down the DE-proteins that [1] respond to both the virulent and avirulent pathogen infections at either 24 or 72 hpi, and [2] belong to the “response to stimulus” GO category. The 40 proteins satisfying these two criteria (Table 1) were regarded as basal defense-related proteins and were pursued for further study. Among the 40 basal defense-related proteins, 18 proteins have functions related to “responses to oxidative stress”, seven proteins are related to “responses to biotic stress”, and four proteins are related to “responses to cold”, respectively. Proteins involved in “responses to salt stress”, “responses to ion stress”, “responses to chemical stress”, and “responses to water stress” were also identified (Table 1 and Figure 1B). Interestingly, more than half of the defense-related proteins (25/40) are related to two principal GO categories: “responses to oxidative stress” (18/40) and “responses to biotic stress” (7/40). These results indicate that after *M. oryzae* infection, these two biological processes are preferentially activated. Therefore, we concentrated our study on proteins from these two biological processes.

**Figure 3:**
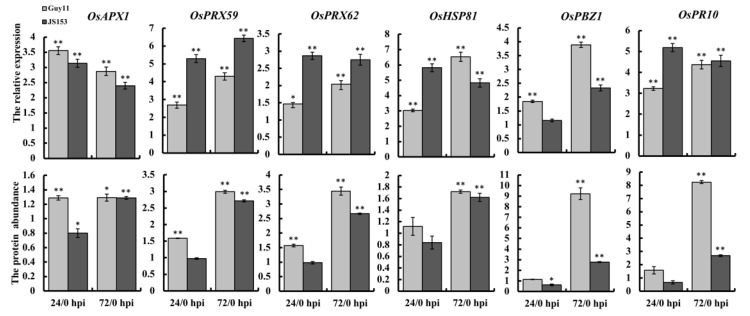
Expression coalition between the transcription and the translation levels. Top: expression of the six selected genes examined by qRT-PCR at the indicated time points by Guy11 infection or JS153 infection. Values represent the means ± SD of three independent samples (* *p* < 0.05, ** *p* < 0.01). Similar results were obtained from three biological repeats. Bottom: protein abundance of the six selected genes. Error bars indicated SD. Asterisks indicate significant differences (* *p* <0.05, ** *p* < 0.01).

We selected three proteins from each category for further validation. From the “responses to oxidative stress” group, we selected OsAPX1 (B7E6Z4), OsPRX62 (Q7XSU7), and OsPRX59 (Q9ST80). From the “responses to biotic stress” group, we selected OsPBZ1 (Q40707), OsPR10 (Q75T45), and OsHSP81 (I1QJW3). Both Guy11 and JS153 infection induced the expression of OsAPX1 at 72 hpi but only by Guy11 infection at 24 hpi. qRT-PCR validation indicated that the transcription level of OsAPX1 was elevated in both Guy11- and JS153-infected rice at 24 and 72 hpi (Figure 3). Similarly, the protein levels of OsPRX59 and OsPRX62 were all induced both by Guy and JS153 infections at 72 hpi but not in JS153-infected rice at 24 hpi. Their transcriptional expression showed durative elevation at both 24 and 72 hpi, no matter whether infected by Guy11 or by JS153 (Figure 3). Our results indicate that the expression of these three proteins involved in responses to oxidative stress is induced by both virulent and avirulent *M. oryzae* strains. OsHSP81, OsPBZ1, and OsPR10 were induced by both Guy and JS153 at 72 hpi. However, the qRT-PCR results agreed with both OsHSP81 and OSPR1 but not OsPBZ1 by JS153 at 72 hpi. In general, our results showed a good coalition between transcription and translation on these proteins (Figure 3).

**Figure 4:**
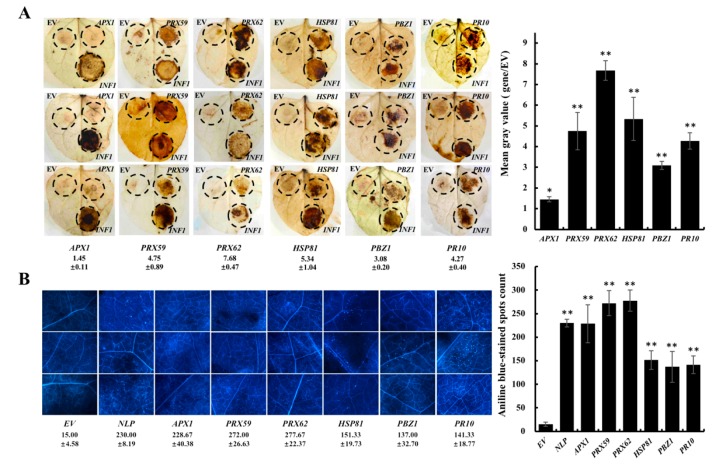
The DEP candidates contribute to callose deposition and ROS accumulation. (**A**) Left: 3,3′-diaminobenzidine (DAB) staining for reactive oxygen species (ROS) accumulation in *N. benthamiana* leaves after infiltration with *A. tumefaciens* carrying empty vector, *PcINF1*, and the six selected genes. The reddish-brown at the injection site shows the accumulation of ROS. Numbers are the relative accumulation and standard deviations of ROS using Image J. Right: Histogram represents the relative accumulation of ROS in the images. Error bars indicate SD from three technical replicates. Asterisks indicate significant differences (* *p* < 0.05, ** *p* < 0.01); (**B**) Left: Aniline blue staining for callose deposition in the leaves (40× magnification) expressing EV (empty vector), *PsNLP* and six selected genes. Numbers are the means and standard deviations of three 1 cm^2^ microscopic fields of view. Right: Histogram represents the means of three 1 cm^2^ microscopic fields of view. Error bars indicate SD. Asterisks indicate significant differences (** *p* < 0.01).

We transiently expressed these proteins in *Nicotiana benthamiana* leaves and examined their ability to induce ROS production. OsAPX1 did not induce discernable ROS production, as manifested by comparable ROS signals between OsAPX1 and the empty vector (EV), as well as by the dramatic difference when compared to PcINF1, a *Phytophthora* effector with known ROS-eliciting function (Figure 4A). In contrast, both OsPRX59 and OsPRX62 induced significant ROS production, which was almost five to eight times more than that in the EV inoculation. OsHSP1 induced around five times more ROS than the EV did. Significant ROS production was also observed in *N. Benthamiana* leaves transiently expressing OsPR10 (4 folds) and OsPBZ1 (about 3-fold).

Callose deposition was also monitored in *N. Benthamiana* leaves when these proteins were transiently expressed. Proteins associated with “responses to oxidative stress” (OsAPX1, OsPRX59, and OsPRX62) induced dramatic callose deposition in all the samples examined, which is comparable to PsNLP, a *Phytophthora* effector eliciting significant callose deposition. The proteins from the “response to biotic stress” category also induced a significant amount of callose deposition when they were transiently expressed in *N. Benthamiana* leaves, but to a lesser degree than OsAPX1, OsPRX59, and OsPRX62 (Figure 4B).

**Figure 5:**
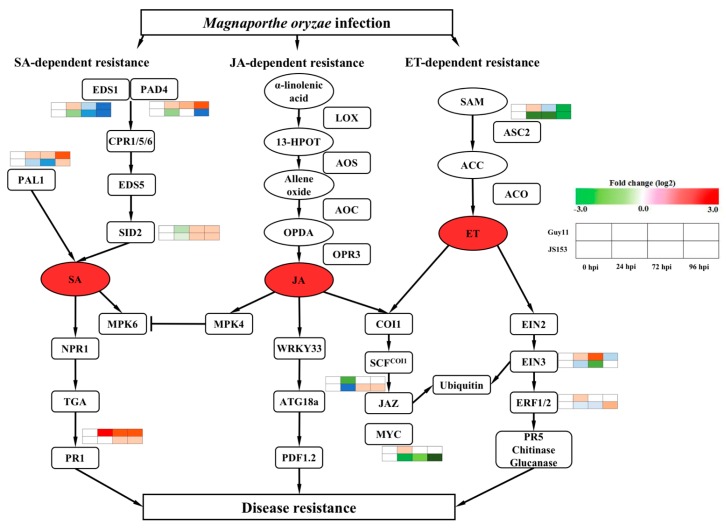
The defense signaling pathway in response to *M*. *oryzae* in rice. Critical components involved in the SA, JA, and ET signaling pathways are analyzed by real-time PCR. Rectangles indicate genes or proteins; ovals indicate chemical compounds, red ovals indicate phytohormones. The chart with different colors indicates the expression of indicated genes at the indicated time points by Guy11 infection or JS153 infection.

Guy11 and JS153 are *M. oryzae* strains with distinct pathogenesis mechanisms and pathogenicity. To demonstrate these two strains can be distinguished by rice and can elicit distinct phytohormone responses, we examined several critical phytohormones along the time course of infection. SA, JA, and ET (ethylene) are critical phytohormones associated with rice innate immunity against blast disease [14,17]. We evaluated the responses of these critical phytohormones after both Guy11 and JS153 infection by examining the expression of selected key components [14]. *OsPAD4* and *OsEDS1* are SA synthetic regulators. *OsPAD4* was induced by Guy11 but not JS153 at 24, 72, and 96 hpi, while *OsEDS1* was suppressed by both pathogens at most of the time points except 24 hpi, which was slightly induced (Figure S1). *OsSID2* is an SA synthetic component, which was induced by both Guy11 and JS153 at 72 and 96 hpi. *OsPAL1* is associated with both SA and lignin synthesis, which was induced only by Guy11 but not JS153. *OsPR1* showed similar induction profile as *OsPAL1* but to a much higher degree (Figure S1). We also examined key components involved in JA synthesis and signaling. *OsJAZ1* was slightly induced by JS153 at 72 and 96 hpi but not by Guy11. *OsMYC2* was suppressed by both Guy11 and JS153 at 72 and 96 hpi while it was only temporarily induced by Guy11 at 24 hpi. These results indicate that the SA and JA signaling is perturbed by *M. Oryzae* infection. Aminocyclopropane carboxylic acid synthase (ACS) is the committed and rate-limiting enzyme in the biosynthesis of ethylene. *OsACS2* was suppressed by both Guy11 and JS153 infection. *EIN3* (*ethylene-insensitive 3*) encodes a nuclear transcription factor that initiates downstream transcriptional cascades for ethylene responses. The expression of *OsEIN3* was suppressed by JS153 and by Guy11 except at 72 hpi. Ethylene Response Factors (ERFs) encode transcription factors that regulate the molecular response to pathogen attacks [18]. We examined the expression of *OsERF1/2* and found that the expression level was marginally altered (Figure S1). Taken together, the SA signaling pathway is induced by Guy11 infection while remains unaffected or even slighted suppressed by the JS153 infection. In contrast, the JA and ET signaling pathways are unaltered or suppressed by either Guy11 or JS153 infection (Figure 5).

## 3. Discussion

Quantitative proteomics greatly prompts biological studies by not only identifying trace amount proteins in the samples but also comparing the relative abundance of proteins among different samples. The 1618 proteins presented in this report were from two independent quantitative proteomic analyses, by which the credibility of our proteomic measurement is guaranteed. DE-proteins were filtered by both confidence (coverage > 10%) and accuracy (*p* < 0.5) so that only a condensed but highly relevant group of proteins were further studied and validated. The agreement between the transcriptional and translational expressions of the six examinees manifests a close coalition between the proteomic data and their in vivo expression levels (Figure 3). In this study, we compared protein expression profiles between healthy rice and rice infected with the virulent (Guy11) and avirulent (JS153) strains. We focused on proteins exhibiting similar responses to both these *M. oryzae* strains, which represent common rice defense responses against both the virulent and avirulent strains. By investigating the general defense machinery to broad-spectrum rice blast disease, we expect to identify common mechanisms that would be useful for developing broad-spectrum blast disease resistance.

Our research strategy is supported by the preferential enrichment of defense-related biological processes shared by rice either infected by Guy11 or by JS153, and the meagerness of such proteins in rice only infected by Guy11 or by JS153 (Figure 2). For example, the “regulation of protein serine/threonine phosphatase activity” biological process is enriched by both Guy11 and JS153 infection. OsPBZ1 and OsPR10 belonging to this biological process have been demonstrated to exhibit fundamental roles in rice basal defense in other genetic studies [19,20,21,22]. Involvement of proteins belonging to the “response to oxidative stress” in plant disease resistance has also been demonstrated in many plant species [23,24]. Other biological processes such as “protein folding” and “glutathione metabolic” have also been linked to plant basal defense [25,26,27,28]. Therefore, our proteomic investigation of rice proteins responding to both Guy11 and JS153 infections will facilitate our understanding of broad-spectrum resistant mechanism.

Of the DE-proteins 19% were associated with “response to stimulus”, an unexpected result, verifying a great enrichment of the defense-related proteins after the blast disease pathogens’ infection. When further narrowed down to stimulus-responding proteins that differentially expressed upon both Guy11 and JS153 infections, the list condensed to 40 proteins (Table 1). Outstandingly, 18 out of the 40 proteins belong to “response to oxidative stress”, and five proteins belong to the “response to biotic stress” category. These two biological processes are well known for their involvement in defense responses to pathogen infections [29]. Therefore, these results further justify that our analysis strategy targeted authentic immune players in response to blast disease.

Our proteomic discovery was further validated by whether the examinees could contribute to consensus basal defense responses such as inducing ROS production and callose deposition. Leaf discs transiently expressing OsAPX1 caused callose deposition but did not accumulate detectable ROS, which is in line with its ROS-scavenger activity in other plants [30]. In contrast, transient expression of OsPRX59 and OsPRX62 induced a significant amount of ROS. OsPRX59 and OsPRX62 are class III plant peroxidases with multiple functions, such as ROS removal and cell wall biosynthesis [31,32]. Significant ROS accumulation upon OsPRX59 and OsPRX62 expression overrules their potential of ROS removal and makes their involvement in cell wall synthesis more plausible. In fact, when callose deposition was checked, expression of OsPRX59 and OsPRX62 caused most extensive callose deposition signals (Figure 4B), suggesting these two proteins participate in cell wall synthesis or reinforcement.

OsHSP81 belongs to the HSP90 family that, in rice, is involved in resistance to insect, bacterial, viral, and fungal pathogen infections [25,26,33,34]. Our results showed that OsHSP81 boosted both ROS production and callose deposition, suggesting a decisive role in response to blast disease. OsPBZ1 is highly conserved in plant species [20], which induces programmed cell death. OsPR10 has also been reported in response to various pathogens in multiple plant species, including rice. Previous genetic studies revealed the involvement of OsPBZ1 and OsPR10 in response to blast disease [19,21,35,36]. However, our study confirmed at the proteomic level that OsPBZ1 and OsPR10 are induced by both virulent and avirulent infection, and showed that OsPBZ1 and OsPR10 are capable of inducing both ROS accumulation and callose deposition (Figure 4).

Our previous studies showed that Guy11 and JS153 caused obvious different disease symptoms on the Nipponbare cultivar [14,15]. The phytohormone experiments further demonstrated the difference between these two strains (Figure 5). In general, JA and ET showed suppressed profiles after Guy11 or JS153 infection. In contrast, SA showed a positive association with defense responses to the blast disease (Figure S1). This is inconsistent with the antagonistic relation between the JA/ET and the SA signaling pathways. In specifics, *OsEDS1* and *OsSID2* showed similar reactions to both Guy11 and JS153 infection, indicating at least part of the SA synthesis is regulated by a common mechanism to both virulent and avirulent blast pathogen. However, *OsPAD4*, *OsPAL1*, and *OsPR1* demonstrated a more preferential response to Guy11, instead of to JS153, indicating that rice may recognize and respond to Guy11 individually. Therefore, the proteomic components revealed in this study represent common rice defense responses against these representational strains, indicating a general defense machinery to broad-spectrum rice blast disease. Specifically, our results indicate that “responses to oxidative stress” and “responses to biotic stress” are critical biological processes to blast disease. Breeding projects concentrated on modification of several critical components such as OsPRX59 and OsPRX62 should foresee the success of resistant rice lines to a broad spectrum of blast disease pathogens.

## 4. Materials and Methods

### 4.1. Plants and Inoculation

Rice (*Oryza sativa* L. japonica. cv. Nipponbare) and *N. benthamiana* were grown in a growth room maintained at 25 °C and 70% relative humidity with a 12-h/12-h light/dark photoperiod. Three-leaf-stage plants were spray-inoculated with gelatin or indicated *M. oryzae* conidial suspensions (1 × 10^5^ spores/mL in 0.2% gelatin) [37]. The inoculated plants were kept in darkness at 80% RH for 24 h before they were transferred to a growth chamber at 25 °C, 80% relative humidity, and a 12-h/12-h light/dark photoperiod.

### 4.2. Protein Extraction

Rice seedlings (0.5 g) were ground to powder in liquid nitrogen and then dissolved in 10% tricarboxylic acid (TCA)/acetone (*w*/*v*) containing 0.1% DL-dithiothreitol (DTT)at −20 °C for 2 h. The supernatant was discarded after centrifugation at 40,000 *g* for 20 min. The pellet was washed twice with cold acetone, lyophilized, and dissolved in 300 μL of a lysis solution containing 7 M urea, 2 M thiourea, 4% *w*/*v* 3-[(3-cholamidopropyl)-dimethylammonio]-1-propane sulfonate (CHAPS), 65 mM DTT, and 1 mM phenylmethanesulfonyl fluoride (PMSF). The proteins were labeled by an iTRAQ 8-plex kit (AB Sciex) and measured by a Triple TOF 5600 mass spectrometer.

### 4.3. Protein Digestion, iTRAQ Labeling, and Strong Cation Exchange

Protein samples (100 μg of each protein) were mixed with dissolution buffer from AB Sciex (Framingham, MA, USA), digested with trypsin at a 20:1 mass ratio at 37 °C for 14 h, then labeled using the iTRAQ Reagents 8-plex kit according to the manufacturer’s instructions (AB Sciex).

The labeled samples were then pooled and dried in an Eppendorf vacuum concentrator. Then, the samples were mixed and lyophilized before dissolving in 4 mL of strong cation exchange (SCX) buffer A (25 mM NaH_2_PO_4_ in 25% acetonitrile, pH 2.7). The peptides fractionated on an Ultremex SCX column (4.6 mm × 250 mm) using an Agilent 1200 HPLC were grouped into ten components. An Exigent Nano LC-Ultra 2D system (AB Sciex) was used for sample separation. A Triple TOF 5600 mass spectrometer and a Nano Spray III Source (AB Sciex) were used to perform mass spectrometer data acquisition.

### 4.4. Database Search and iTRAQ Quantification

ProteinPilot™ software (version 4.2) was used for raw data processing against the database of *Oryza sativa* from UniProt (http://www.uniprot.org). The primary database search parameters were as follows: the instrument was TripleTOF 5600, iTRAQ quantification, cysteine modified with iodoacetamide; and biological modifications were selected as ID and trypsin digestion. Peptides with a global false discovery rate (FDR) <1% were used for further protein annotation. To minimize the incidence of false positives, a strict cutoff of unused ProtScore >1.3 was applied for protein identification.

### 4.5. Gene Ontology Analysis and Biological Processes Analysis

Differentially expressed proteins were classified according to GO analysis in Protein Information Resource (https://pir.georgetown.edu). DAVID (https://david.ncifcrf.gov) was used to predict biological processes.

### 4.6. RT-PCR Analysis of the Small RNAs and Predicted Targets

Total RNA was extracted using the Trizol method, reverse-transcribed using a reverse transcription kit (Takara, Shiga, Japan), and the expression levels of the genes of interest were detected using a Real-time PCR Kit (Takara). Primers used for real-time PCR amplification are listed in Table S5.

### 4.7. Transient Expression Analysis in N. Benthamiana

Transient co-expression assays in *N. benthamiana* were performed by infiltrating 3-week-old *N. benthamiana* leaves with *Agrobacterium* GV3101 (OD_600_ = 0.8) harboring constructs containing the gene of interest (pEG104), or an empty vector as a control [38]. Leaf tissue was collected 48 hpi, and protein expression was detected by Western blot.

### 4.8. DAB Staining

*N. benthamiana* leaves were placed in 1 mg/ml of 3,3′-diaminobenzidine (DAB) (Sigma-Aldrich, St. Louis, MO, USA) and shaken at 26 °C for 8 h in a dark place. Then the leaves were decolorized in a 94:4 ethanol:acetic acid (*v*/*v*) solution at 26 °C for 8 h in dark place. Stained leaf was observed under a camera [39].

### 4.9. Aniline Blue Staining

Leaves were fixed in 94:4 ethanol:acetic acid (*v*/*v*) solution for 8 h and then stained with 1 mg/mL aniline blue in 150 mM sodium phosphate (pH = 7.0) in the dark for more than 1 h at room temperature (25 °C). Stained leaves were mounted using 50% glycerol. Callose was observed under a microscope (Olympus, Tokyo, Japan) [40,41].

## Figures and Tables

**Table 1:** Significant rice differentially expressed (DE)-proteins responding to *M. oryzae.*

Protein ID	Name	Annotation	Guy11		JS153	
			24/0 hpi	72/0 hpi	24/0 hpi	72/0 hpi
response to oxidative stress						
Q652L6	MDAR3	cellular oxidant detoxification	1.20	1.33	0.85	1.35
B7E6Z4	APX1	hydrogen peroxide catabolic process	1.29	1.29	0.80	1.29
I1Q8M5	TRXh1	oxidoreductase activity	1.11	1.35	0.95	1.47
A0A0E0Q2V5	CATB	hydrogen peroxide catabolic process	1.17	1.81	0.88	1.86
Q7XSU7	PRX62	hydrogen peroxide catabolic process	1.57	3.44	0.98	2.67
Q9ST80	PRX59	hydrogen peroxide catabolic process	1.59	2.99	0.98	2.72
Q5U1T0	PRX13	hydrogen peroxide catabolic process	1.55	2.70	0.91	3.05
Q5Z4D3	PRX78	hydrogen peroxide catabolic process	1.44	1.71	0.75	1.72
Q5Z7J2	PRX86	hydrogen peroxide catabolic process	1.71	2.04	1.14	1.73
Q654S1	PRX12	hydrogen peroxide catabolic process	1.42	1.54	0.96	1.82
Q6AVZ8	PRX65	hydrogen peroxide catabolic process	1.26	1.41	0.78	1.35
Q6ESJ0	GPX3	glutathione peroxidase activity	1.15	1.36	0.93	1.58
Q6EUS1	PRX27	hydrogen peroxide catabolic process	1.35	1.56	0.84	1.79
Q6Z4E4	ALDH6B2	methylmalonate-semialdehyde dehydrogenase activity	1.27	1.66	0.87	1.82
Q7XHB3	PRX125	hydrogen peroxide catabolic process	1.39	1.77	1.02	1.75
Q9FEV2	riPHGPX	glutathione peroxidase activity	1.36	1.75	0.8	1.54
Q8W317	NADH dehydrogenase	NADH dehydrogenase activity	1.25	1.37	0.79	1.53
A0A0E0PU51	alkaline α-galactosidase	catalytic activity	1.54	1.62	1.02	1.83
**response to biotic stimulus**						
Q7XPU1	Harpin-induced 1 domain containing protein	signal transducer activity	1.34	1.50	0.93	1.47
Q40707	PBZ1	response to biotic stimulus, defense response	1.14	9.22	0.63	2.77
I1QJW3	HSP81	response to stress, ATP binding	1.12	1.72	0.84	1.62
Q75T45	RSOsPR10	pathogenesis-related protein	1.58	8.25	0.66	2.68
Q75L45	OsRLCK178	cell surface receptor signaling pathway	1.49	1.55	1.02	1.66
A0A0E0PMK8	OsGDI1	protein transport	1.17	1.6	0.77	1.37
Q945E9	JIOsPR10	response to biotic stimulus	1.92	2.55	1.03	2.19
**response to cold, salt stress, water deprivation**						
Q8LHG8	Os01g0542000	isomerase activity	1.35	1.53	0.87	1.56
I1QGF2	YchF1	hydrolyzes ATP	1.22	1.31	0.85	1.37
Q7XXS0	RMtATPd2	mitochondrial membrane ATP synthase	1.29	1.46	0.92	1.41
Q7XUC9	Histone H4	transcription regulation, DNA repair, DNA replication	0.96	1.44	0.91	1.32
I1PYW0	Os6PGDH1	phosphogluconate dehydrogenase activity	1.36	1.70	0.89	1.59
I1PUR5	UspA	response to stress	3.26	1.18	2.17	1.38
**response to ion stress**						
Q5JK10	Os01g0926300	response to cadmium ion	1.15	1.59	0.98	1.52
Q6H734	Os02g0198600	ubiquitin binding	1.35	1.53	0.87	1.50
S4TZU3	Os02g0621700	magnesium ion binding	1.23	1.49	0.79	1.31
**response to chemical**						
Q5W676	HXK5	fructose and glucose phosphorylating enzyme	1.06	1.27	0.79	1.43
Q2QYK6	chalcone isomerase	chalcone isomerase activity	1.15	1.4	0.94	1.59
Q852M0	GDH1	glutamate dehydrogenase activity	1.37	1.49	0.83	1.45
Q8S718	OsGSTU23	glutathione transferase activity	1.28	1.73	0.78	1.68
**response to physical stress**						
Q10LV7	LOC_Os03g21560	cellular response to light intensity	1.20	1.36	0.81	1.44
Q7XRB6	Os04g0435700	response to UV-B, photoreceptor activity	1.39	1.46	0.75	1.46

## Supplementary Materials

Supplementary materials can be found at http://www.mdpi.com/1422-0067/19/7/1950/s1.
