# Supplementary material for: Quantitative Proteomic Analysis Provides Insights into Rice Defense Mechanisms against Magnaporthe oryzae
Source: Int J Mol Sci. 2018 Jul 3;19(7):1950. doi: 10.3390/ijms19071950 (PMC6073306; doi:10.3390/ijms19071950)
Supplement: Supplementary file 1 [file ijms-19-01950-s001.zip › Supplementary Files/Supplemental Information (1).docx]

**Supplemental Information**

**Figure S1. *M. Oryzae* infection perturbs the SA and JA signaling**.

Relative expression of SA, JA, and ET signaling pathway-related genes at the indicated time points upon Guy11 infection or JS153 infection. Values represent the means ± SD of three independent samples (**P*＜0.05, ***P*＜0.01). Similar results are obtained from three biological repeats.

**Table S1.** Summary of rice proteins identified in two independent quantitative proteomic analysis;

**Table S2.** Summary of consensus rice proteins identified by both quantitative proteomic analysis;

**Table S3.** Summary of rice DE-proteins;

**Table S4.** Primers information.
