# Supplementary figures and images for: Quantitative Proteomic Analysis Provides Insights into Rice Defense Mechanisms against Magnaporthe oryzae
Source: Int J Mol Sci. 2018 Jul 3;19(7):1950. doi: 10.3390/ijms19071950 (PMC6073306; doi:10.3390/ijms19071950)

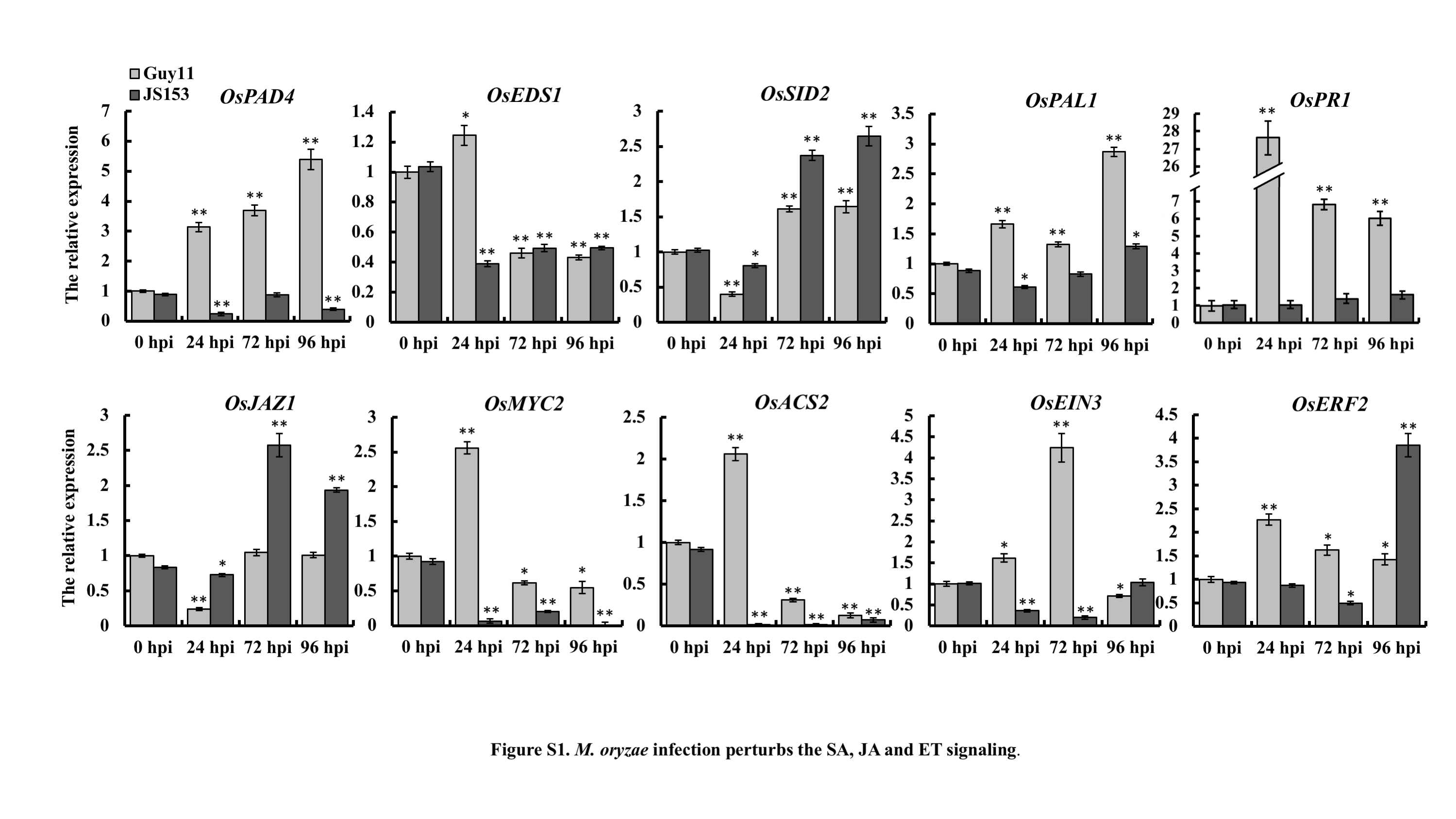

Supplement: Supplementary file 1 [file ijms-19-01950-s001.zip › Supplementary Files/Figure S1.png]
